# Senna Laxative-Associated Severe Ischemic Bowel Disease in an 87-Year-Old Patient: A Clinical Warning

**DOI:** 10.7759/cureus.105910

**Published:** 2026-03-26

**Authors:** DI He, Lin Liu, Xiangmei Duan, Wenyong Shen, Jianhua Shi

**Affiliations:** 1 Internal Medicine Department, Fuling Hospital Affiliated to Chongqing University, Chongqing, CHN; 2 Gastroenterology Department, Fuling Hospital Affiliated to Chongqing University, Chongqing, CHN

**Keywords:** anthraquinone, ischemic colitis, laxative abuse, senna laxative, senna side effects

## Abstract

Senna laxative, one of the major stimulant laxatives, has received less attention regarding its potential risk of intestinal ischemia in the elderly population. An 87-year-old female with a past medical history of chronic constipation developed abdominal pain and fever after oral ingestion of senna laxative for constipation and was subsequently diagnosed with ischemic bowel disease upon hospitalization. Symptomatic and supportive treatment, such as antibiotics, acid inhibition and stomach protection, fluid replenishment, and intravenous nutrition, was given. After treatment, the patient's condition stabilized, her symptoms improved, and she was allowed to leave the hospital. This case highlights that the use of senna laxative in elderly patients may lead to severe ischemic intestinal disease. Clinicians should be vigilant about the adverse reactions of senna laxative in elderly patients.

## Introduction

Ischemic colitis is the most common type of gastrointestinal ischemia, accounting for approximately 50-60% of all cases [1]. It results from a reduction in blood flow to a level that is inadequate for the delivery of nutrients and oxygen to the intestinal tissue. It can occur in everyone, but it is most common in the elderly (80%) and women (76%). The risk factors for its onset include hypertension (58%), diabetes (24%), cardiovascular diseases (23.8%), kidney disease (15%), and atrial fibrillation (14.3%). The mortality rate is higher in severe cases [2]. The overall mortality rate in large series ranges from 4% to 12%, and the recurrence rates can be as high as 15% in three years [3]. Ischemic colitis is thought to be multifactorial, and its clinical severity can range from mild to severe, the latter of which is associated with intestinal gangrene and may require surgical intervention. Here, we present a case of ischemic colitis secondary to senna use.

## Case presentation

An 87-year-old Chinese female had a history of chronic constipation, hypertension, and coronary artery disease (CAD) and denied having atrial fibrillation or severe peripheral arterial sclerosis. She was admitted to our hospital following the onset of persistent, colicky abdominal pain and abdominal distension one day prior. The symptoms occurred after oral ingestion of 10 g of senna laxative for constipation relief (no bowel movement for one week). Despite passing a small amount of formed yellow stool, her symptoms persisted and were later accompanied by chest tightness, exertional dyspnea, and chills with fever (peak temperature: 38.9°C) within 12 hours, prompting hospitalization.

Her vital signs were as follows: blood pressure of 128/76 mmHg, heart rate of 98/minute, respiratory rate of 20/minute, temperature of 38.9°C, and oxygen saturation 100% on room air. The patient appeared acutely ill with a distressed facial expression and exhibited lethargy (impaired mental status). Abdominal examination revealed distension, mild rigidity on palpation, and diffuse tenderness. Murphy's sign was equivocal, raising suspicion for possible positivity. Bowel sounds were normoactive. Laboratory values showed lactic acid (3.10 mmol/L), D-dimer (4.09g/L), and white blood cells (15.64 x 109/L) (Table 1).

**Table 1:** Laboratory values

Lab test	Value	Reference range
White blood cells	15.64×10^9^/L	3.50-9.50×10^9^/L
Hemoglobin	117 g/L	115-150 g/L
Blood urea nitrogen	7.50 mmol/L	2.50-7.10 mmol/L
Creatinine	64.0 umol/L	40.0-100.0 umol/L
Albumin	31.5 g/L	10.0-55.0 g/L
Procalcitonin	2.670 ng/ml	<0.05 ng/mL
Lactic acid	3.10 mmol/L	0.50-2.20 mmol/L
D-dimer	4.09 mg/L	0.00-0.55 mg/L

**Figure 1:**
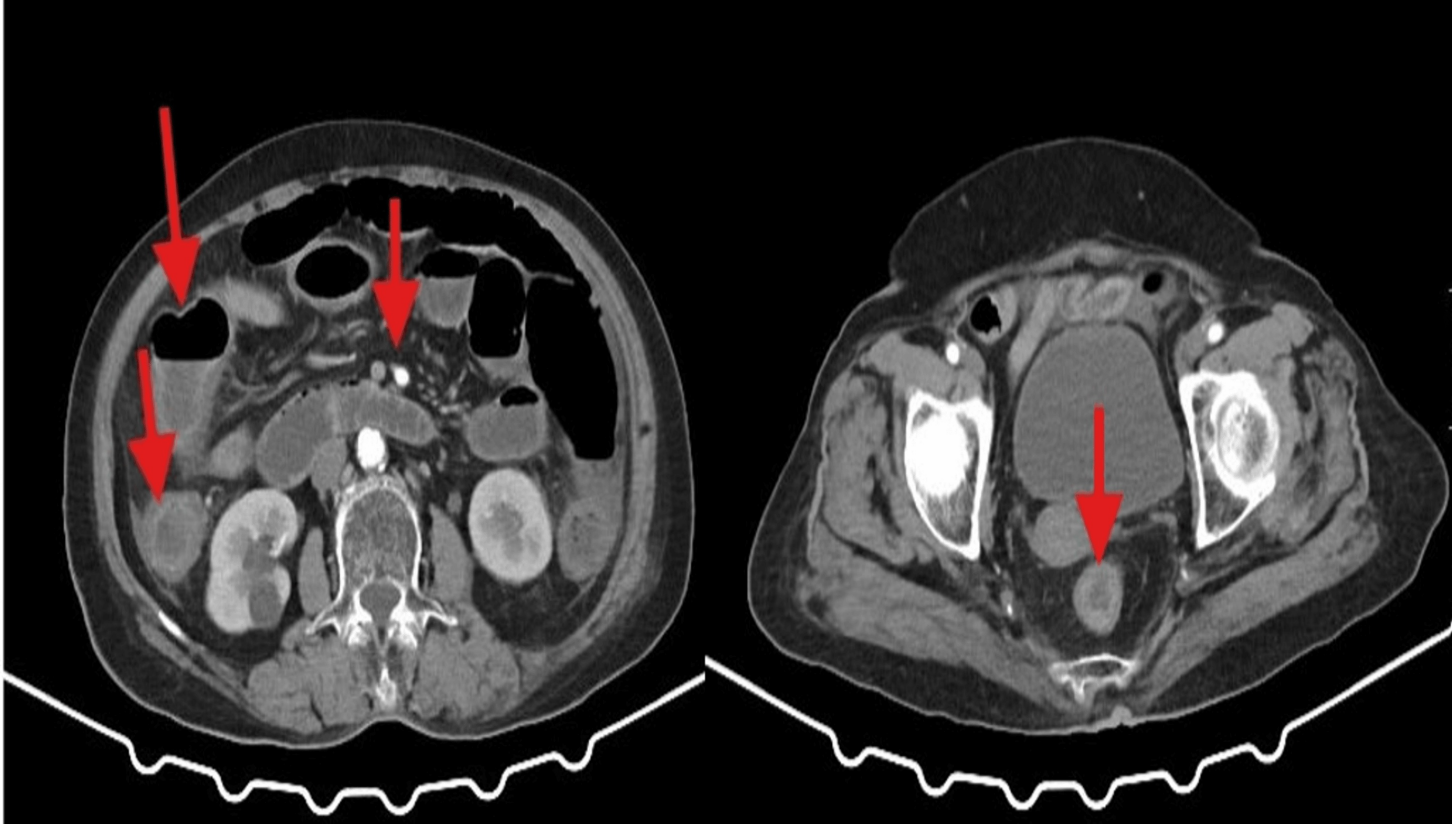
Axial view computed tomography with intravenous contrast Multiple calcified plaques in the superior mesenteric artery, thickening and edema of the intestinal walls of the ascending colon and transverse colon, incomplete low-position small intestinal obstruction, and thickening of the intestinal wall in the lower segment of the rectum (indicated by the red arrow).

Contrast-enhanced computed tomography of the abdomen and pelvis demonstrated multiple calcified plaques in the abdominal aorta and superior mesenteric artery, thickening and edema of the intestinal walls of the ascending colon and transverse colon, incomplete low-position small intestinal obstruction, and thickening of the intestinal wall in the lower segment of the rectum (Figure 1).

**Figure 2:**
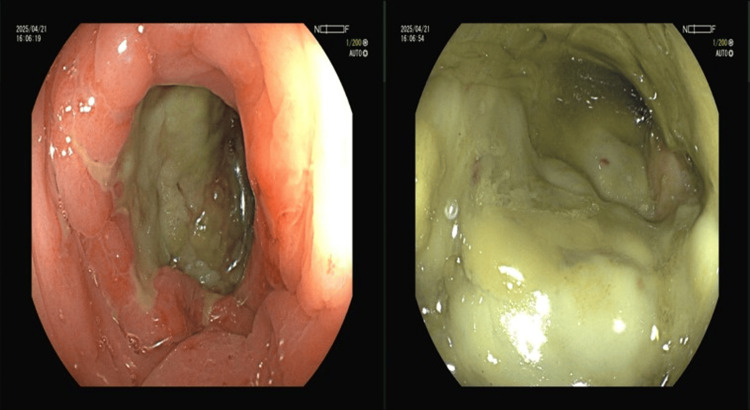
The first colonoscopy examination result indicated that the mucosa of the sigmoid colon is congested and swollen, with deep ulcerations and a thick yellowish coating on the surface

A colonoscopy was performed two days after admission. which showed changes consistent with ischemic colitis at a distance of 18 cm from the anus, a deep ulceration ring approximately one full circle in circumference appeared at the proximal end, with a yellowish-white coating on the surface and significant swelling; no obvious mucosal congestion or erosion was observed at the distal end. The scope was no longer advanced due to the ischemic changes seen and poor bowel preparation (Figure 2).

**Figure 3:**
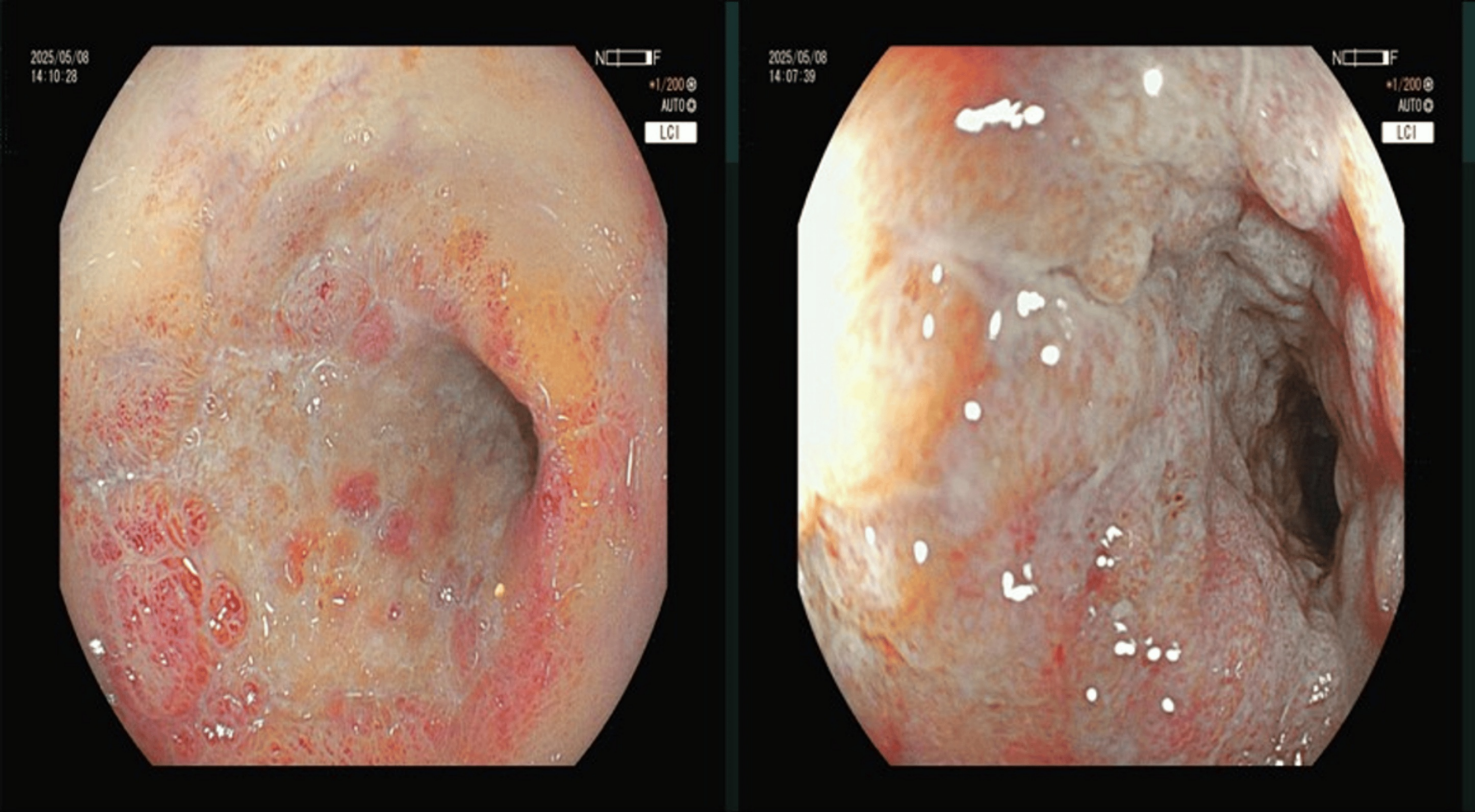
Colonoscopy findings conducted 17 days after the first one The lumen of the sigmoid colon is deformed, with hyperplasia of granulation tissue and formation of fibrous scars observed, but no white coating is present.

On the first day of the patient's hospitalization, due to suspected intestinal ischemia, imipenem-cilastatin was administered for anti-infection treatment, while low molecular weight heparin 2,500 units was given for preventive anticoagulation therapy. Papaverine was used to dilate blood vessels, and 2,000 mL of crystalloid fluids were replenished. Three days later, the patient still had a fever with a body temperature fluctuating between 38.0 and 38.5℃. The abdominal pain and abdominal distension did not show significant improvement. After treatment with broad-spectrum antibiotics, including imipenem-cilastatin and linezolid, the patient's symptoms were relieved. On the seventh day of hospitalization, white *Candida *was cultured from the patient's stool, and nystatin was added for antifungal treatment. On the 19th day of hospitalization, the colonoscopy findings showed that the sigmoid colon ulcer had healed, with visible scar formation and narrowing of the lumen (Figure 3), and the condition had significantly improved. The patient then recovered and was discharged.

## Discussion

Senna laxative is commonly used herbal laxatives and classiﬁed as stimulative laxatives. The anthraquinones is the effective constituents [4], Which is hydrolyzed by intestinal bacteria to produce stimulating metabolites. These metabolites can stimulate the contraction of intestinal smooth muscle cells and increase intestinal peristalsis. At the same time, they can regulate water channel proteins and reduce the expression level of AQP3 in the colon, thereby regulating the water transport and achieving the laxative effect [5]. Common side effects include abdominal pain, diarrhea, electrolyte imbalance (hypokalemia), and disorder of the intestinal microbiota，chronic use-induced colon melanosis and laxative dependence [6]. Furthermore, research has shown that senna laxative can damage the integrity of the intestinal mucosal barrier, leading to chronic low-level inflammation in the colon [7] and promoting the development of colon cancer [8]. However, up to now, no literature reports have been found regarding the occurrence of ischemic bowel disease caused by senna laxative. The exact pathogenic mechanism is still unclear, but it is mainly considered to be related to the following factors.

First, strong intestinal contractions cause compression of blood vessels. Senna laxative can directly stimulate the plexus of the colonic myenteric nerve and facilitate colon peristalsis [7]. However, this kind of peristalsis is intense and uncoordinated, which may cause intestinal spastic contractions and increased intestinal pressure, and the small blood vessels within the intestinal wall (especially the straight small vessels) may be compressed and occluded, directly causing local ischemia. This elevation in intraluminal pressure was likely a contributing factor to the development of ischemic colitis that she sustained.

Second, gastrointestinal motility disorder and ischemia-reperfusion injury: Abnormally strong peristalsis can increase the pressure in the intestinal lumen and reduce the capillary perfusion pressure, and the disorder of contraction and relaxation may induce microcirculation disorders. When spasm relief or the effect of the drug weakens, reperfusion of blood flow may initiate the ischemia-reperfusion injury process, generating a large amount of oxygen-free radicals, leading to tissue oxidative damage, increased inflammation, and increased vascular permeability. This may be one of the important mechanisms for the observation of intestinal wall gas accumulation (gas sign) in this case.

Third, potential effects on autonomic nervous function: Anthraquinone compounds may interfere with intestinal nerve regulation and affect vascular tension. Secondary dehydration/low blood volume: Severe diarrhea can lead to a reduction in effective circulating blood volume, further lowering the intestinal perfusion pressure, which becomes an important contributing factor in triggering or exacerbating ischemia. It is believed that the colon is particularly susceptible to ischemia owing to its relatively low blood flow, lack of collateral perfusion, its unique decrease in blood flow during periods of functional activity, and its sensitivity to autonomic stimulation.

The special susceptibility of elderly patients and hypertension in this case cannot be ignored. Advanced age and hypertension are independent risk factors for ischemic bowel disease. Elderly patients often have age-related arteriosclerosis and microcirculation dysfunction, and the reserve capacity of mesenteric vessels and their adaptability to changes in blood flow significantly decline. Therefore, in this case, an elderly woman developed severe ischemic bowel disease without any clear underlying serious vascular disease, which strongly suggests the high risk of using senna laxative in the elderly population.

During the diagnosis process, we carefully ruled out other possible causes that might have led to similar symptoms. The patient had no history of an unhygienic diet or exposure to cold, which did not support infectious colitis. There was no history of inflammatory bowel disease (IBD) in the past, and the clinical symptoms and colonoscopy findings did not match those of a typical acute attack of IBD. Contrast-enhanced computed tomography did not show occlusion of the main arteries or veins of the mesentery, and the patient had no atrial fibrillation, severe heart failure, or known hypercoagulable state, which reduced the possibility of occlusive vascular diseases. Therefore, we speculated that senna laxative was the specific exogenous factor that triggered colonic ischemia.

This case provides an important warning for clinical practice: Stimulant laxatives, such as senna laxative, have the potential risk of inducing ischemic bowel disease. Before use, the patient's age and underlying conditions should be fully considered. For elderly patients with constipation, a personalized laxative regimen should be developed. Osmotic laxatives (e.g., polyethylene glycol) are generally considered relatively safe options [9]. When treating elderly patients presenting with acute abdominal pain, especially those with known constipation and a history of using laxatives, a detailed inquiry about the history of laxative use should be conducted, and drug-induced ischemic bowel disease should be taken into consideration.

## Conclusions

In conclusion, this case report emphasizes that the use of senna laxative in elderly patients may lead to severe ischemic intestinal disease. The mechanism may be related to drug-induced severe intestinal spasms, compression of blood vessels, ischemia-reperfusion injury, and dehydration. Moreover, the physiological vascular changes and reduced compensatory ability in elderly patients significantly increase this risk. Clinicians need to be highly vigilant of this rare but serious complication, carefully evaluate the risk-benefit ratio of using irritating laxatives in elderly patients, and provide adequate medication guidance and monitoring.

## References

[1] An Q, Yuan B, Guo Z (2022). Clinical characteristics and long-term outcomes of hospitalised patients with ischemic colitis with different degrees of haematochezia: a retrospective study. Eur J Gastroenterol Hepatol.

[2] Maimone A, De Ceglie A, Siersema PD, Baron TH, Conio M (2021). Colon ischemia: a comprehensive review. Clin Res Hepatol Gastroenterol.

[3] Demetriou G, Nassar A, Subramonia S (2020). The pathophysiology, presentation and management of ischaemic colitis: a systematic review. World J Surg.

[4] Lombardi N, Bettiol A, Crescioli G (2020). Association between anthraquinone laxatives and colorectal cancer: protocol for a systematic review and meta-analysis. Syst Rev.

[5] Wang Z, Cheng Y, Su W (2021). Organ specific differences in alteration of aquaporin expression in rats treated with sennoside A, senna anthraquinones and rhubarb anthraquinones. Int J Mol Sci.

[6] Chiba T, Wang T, Kikuchi S (2024). Colonoscopic resolution of melanosis coli after cessation of senna laxative use. Int Med Case Rep J.

[7] Zhang R, Huang C, Wu F (2023). Review on melanosis coli and anthraquinone-containing traditional Chinese herbs that cause melanosis coli. Front Pharmacol.

[8] Lombardi N, Crescioli G, Maggini V (2022). Anthraquinone laxatives use and colorectal cancer: a systematic review and meta-analysis of observational studies. Phytother Res.

[9] Luo M, Li W, Wang X, Zhou Y (2025). Oral bowel cleansers and ischemic colitis risk: a real-world disproportionality analysis. PLoS One.

